# The Importance of Cancer Registry Linkage for Studying Rare Cancers in Prospective Cohorts

**DOI:** 10.1155/2020/2895276

**Published:** 2020-11-25

**Authors:** Emily Maplethorpe, Emily V. Walker, Trenton Smith, Faith G. Davis, Yan Yuan

**Affiliations:** School of Public Health, University of Alberta, 3-300 ECHA 11405 – 87 Ave, Edmonton, Canada T6G 1C9

## Abstract

Large prospective cohort studies may offer an opportunity to study the etiology and natural history of rare cancers. Cancer diagnoses in observational cohort studies are often self-reported. Little information exists on the validity of self-reported cancer diagnosis, especially rare cancers, in Canada. This study evaluated the validity of self-reported cancer diagnosis in Alberta's Tomorrow Project (ATP), a provincial cohort in Canada. ATP data were linked to the Alberta Cancer Registry (ACR). The first instance of self-reported cancer in a follow-up survey was compared to the first cancer diagnosis in the ACR after enrollment. The sensitivity and positive predictive value (PPV) were estimated for the reporting of cancer status, reporting of common or rare cancer, and reporting of site-specific cancer. Logistic regression analysis explored factors associated with false positive, false negative, and incorrect cancer site reporting. In the 30,843 ATP participants who consented to registry linkage, there were 810 primary cancer diagnoses in the ACR and 959 self-reports of first cancer post-enrollment, for a cancer status sensitivity of 92.1% (95% CI: 90.0-93.9) and PPV of 77.8% (95% CI: 75.0-80.4). Compared to common cancers, rare cancers had a lower sensitivity (62.8% vs. 89.6%) and PPV (35.8% vs. 84.5%). Participants with a rare cancer were more likely to report an incorrect site than those with a common cancer. Rare cancers were less likely to be captured by active follow-up than common cancers. While rare cancer research may be feasible in large cohort studies, registry linkage is necessary to capture rare cancer diagnoses completely and accurately.

## 1. Introduction

Rare cancers account for approximately 25% of cancer cases in Canada and contribute disproportionately to cancer-related morbidity and mortality [1, 2]. However, several challenges including small sample size, diagnostic uncertainty, a lack of knowledge and expertise, and high costs impede progress in the study of rare cancers with conventional clinical research designs [3–5]. Large observational cohort studies, such as the Canadian Partnership for Tomorrow's Heath (CanPath), offer an opportunity to study the etiology and natural history of rare diseases with a sufficiently large sample size [5, 6]. CanPath is a collaboration effort between six regional cohorts, covering 9 of the 10 Canadian provinces. It is Canada's largest volunteer research participant cohort, with over 330,000 participants enrolled to date [7].

Information in large observational cohort studies is often self-reported. Self-reported cancer status should be valid to produce useful results. If self-reported data are not valid, cohort linkage to population-based registries is a necessary step to utilize cohort data for rare cancer research. If self-reported data are deemed valid, then the cohort may be used for etiologic research in the absence of cancer registry linkage.

Most relevant in the evaluation of the validity of self-reported cancer diagnosis is the sensitivity and positive predictive value (PPV). Both a low sensitivity (high false negatives) and a low PPV (high false positives) imply a high likelihood of disease status misclassification and have the potential to bias the results of a study. Specificity and negative predictive value (NPV) are consistently very high in cancer self-report validation studies (both >90%) [8] due to a low cancer prevalence rate; most people do not get cancer, and there are many true negatives relative to false positives and false negatives.

Little information exists on the validity of such self-reports in a Canadian context. Self-report validation studies from the US, Australia, and Europe have found overall sensitivities ranging from 57.5% to 90.3% [8–14] and overall PPV's ranging from 54.9% to 75% [8, 9, 11–14]. The sensitivity and PPV of self-reported cancer diagnosis vary by cancer site [8–14]. A review of the literature did not yield any report that explored whether there was a difference in sensitivity of common versus rare cancers collectively. Age, sex, education, family history of cancer, smoking status, race, time since diagnosis, and comorbidities have been shown to be associated with the validity of self-reported cancer status [8, 10, 12, 13].

To inform rare cancer researchers considering the CanPath cohort, we conducted a study to investigate the sensitivity and PPV of self-reported primary cancer diagnoses and factors associated with self-report validity. Cross-provincial data sharing agreements currently restrict the linkage of CanPath to the Canadian cancer registry for the use of researchers [7, 15]. Thus, our study was confined to data from one of the six regional cohorts that make up the CanPath, Alberta's Tomorrow Project (ATP), where the authors are based.

## 2. Methods

### 2.1. Study Population

The Alberta Tomorrow Project (ATP) is a cohort established in the Canadian province of Alberta, enrolling volunteers between the ages of 35 and 69 with no history of cancer, except nonmelanoma skin cancer [16]. Participants for this study were recruited in Phase 1 of ATP's recruitment, from 2000 to 2008, via random digit dialing [17]. There were 31,203 participants who completed the baseline Health and Lifestyle Questionnaire (HLQ) at the time of their enrolment. There were 360 participants that did not consent to data linkage (i.e., did not provide personal health care number) and were excluded from this study.

### 2.2. Data Sources

Data was obtained from ATP and the Alberta Cancer Registry (ACR). Self-reported cancer diagnosis was collected from the first ATP follow-up survey it was reported: Survey 2004 (S04), Survey 2008 (S08), Updated Health and Lifestyle Questionnaire 2009-2011 (UHLQ), or CORE 2011-2015 (CORE) [16]. These surveys also collected personal and lifestyle information, such as education, smoking status, family health history, place of birth, and other health factors [16].

Alberta Cancer Registry data was linked to ATP data before dispensing the data to the study team. ACR and ATP data were matched on personal health care number and confirmed on first name, last name, and date of birth [16]. Records are linked when a perfect match is found. The ACR is a population-based registry that records topography, morphology, and behavior using ICD-O-3. The ACR has achieved a gold standard from the North American Association of Central Cancer Registries (NAACCR) for complete, accurate, and timely data for many years [18]. Cancer diagnoses that are not mandated to be reported to the Canadian Cancer Registry (CCR), such as behavior 2 cervix and prostate cancers and nonmelanoma skin cancers [19], were excluded to facilitate using the results to explore the utility of linkage to the CCR. Cancer diagnoses with behavior code 0 (benign) or 1 (borderline) diagnoses were also excluded as these were generally not considered “cancers.” In an exploratory analysis, benign and borderline tumors accounted for less than 3% of all registry diagnoses and less than 40% were self-reported. Ethics approval was obtained from the Health Research Ethics Board of Alberta (Study ID CC-16-0880).

### 2.3. Cancer Classifications

ACR cancer site was generated from ICD-O-3 topography codes, using cancer site categories from the Surveillance, Epidemiology, End Results Program (SEER) 2018 classification scheme [20]. Categories that we did not expect to be differentiated in self-reports were collapsed: (1) corpus uteri and uterus, NOS (not otherwise specified) were collapsed to a single “Uterus” category; and (2) oropharynx, nasopharynx, hypopharynx, and pharynx were collapsed into a single “Throat” category. Only ACR diagnoses that occurred within an individual's follow-up time were included, as this diagnosis would have had the opportunity to be reported in their survey(s).

The SO4, SO8, and UHLQ surveys asked participants to record the cancer type in open text. The CORE survey had a dropdown menu to select from a list of cancers corresponding to the SEER site categories and an open text option for other types. Skin cancer responses that did not specify “melanoma” were considered nonmelanoma skin cancer and not included as a self-reported cancer diagnosis. The first instance of self-reported cancer type was categorized into an appropriate site category from the SEER 2018 scheme. SEER cancer categories and corresponding self-report site are described in Supplementary Table S1. Two analysts (EM and TS) independently categorized the first instance of self-reported cancer-type from each participant into a SEER 2018 category with 99.3% (95% CI: 98.9, 99.6%) agreement. Disagreements on cancer type were resolved by consensus.

Through ACR linkage, we identified 118 participants who had a cancer diagnosis prior to their enrollment in ATP. Considering the intention of ATP to enroll only those without a history of cancer, analyses were carried out with and without these 118 participants who had prior cancer diagnoses to see whether these participants may affect self-report validity of incident cancer diagnoses.

### 2.4. Measure of Accuracy

Rare sites were defined as those that had an age-standardized incidence rate < 15/100, 000/year in Canada based solely by site in a recent analysis [1]. Colon cancers, blood/hematopoietic/bone marrow cancers, and lymphatic cancers were grouped together for this analysis; these three site categories were common. Cancer diagnoses in the ACR were used as the gold standard for estimating sensitivity and PPV. Sensitivity was defined as the number of true positives divided by the number of true positives (TP) and false negatives (FN). PPV was defined as the number of true positives divided by the number of true positives and false positives. Sensitivity and PPV were estimated for any-cancer overall diagnosis, common or rare cancer site, and site-specific for each cancer site. The definitions of terms for each analysis can be found in Supplementary Table S2.

### 2.5. Data Analysis

Logistic regression was used to examine the factors associated with three outcomes: (1) incorrectly reporting a cancer diagnosis (FP), (2) failing to report a cancer diagnosis (FN), and (3) failing to report the correct cancer site. Covariates included sex, education, smoking status, family history, place of birth, and age (at cancer report for the first and third outcome and at last follow-up for the second outcome). ACR cancer diagnosis (common or rare) was also included as a covariate for the second and third outcomes. The most recently reported smoking status and family history prior to or at the time of cancer reporting were used. Data on place of birth was not collected at baseline, and so was only investigated in those who took S08, UHLQ, or CORE and reported this information. Covariates in univariate analyses with a *p* value <0.2 were included in the multivariable analysis for each outcome, and multivariable results were reported. All analyses were conducted using STATA IC version 15 [21].

## 3. Results

### 3.1. Sensitivity and PPV of Self-Reported Cancer Diagnoses

In the 30,843 ATP participants who consented to registry linkage, there were 3,187 primary cancer diagnoses in the ACR by 2018, of which 510 were rare cancers as defined in this report. There were 810 diagnoses that occurred during active participant follow-up time and included in this analysis. The large difference in ACR cancer cases and cases included in the study is mainly due to loss to follow-up.  Table 1 summarizes the number of participants that completed each follow-up survey. For example, only 25% of participants responded to the CORE survey. Most participants with an incident cancer did not complete any follow-up survey after their diagnosis. Thus, they were lost to follow-up, and their diagnosis cannot be used in this validation study.

Of the 810 diagnoses included in the study, 724 were common cancer and 86 were rare cancer. There were 959 participants who self-reported a cancer during follow-up (excluding nonmelanoma skin reports) of which 746 were true positives, for an overall sensitivity of 92.1% (95% CI: 90.0-93.9) and PPV of 77.8% (95% CI: 75.0-80.4) (Table 2). Of the 959 self-reports, 768 reported a common cancer, 151 reported a rare cancer, and 40 had an indeterminate cancer site (response missing/do not know, unclear/unspecific, or containing only non-site-specific histological information). Reporting a common cancer had a sensitivity and PPV of 89.6% (95% CI: 87.2-91.8%) and 84.5% (95% CI: 81.7-87.0%), respectively. Reporting a rare cancer had a much lower sensitivity and PPV of 62.8% (95% CI: 51.7-73.0%) and 35.8% (95% CI: 28.1-44.0%), respectively. Since instruction to not report nonmelanoma skin cancer changed across surveys and skin cancer had fairly low sensitivity and PPV compared to other common cancers, the sensitivity and PPV of common cancer were also calculated without skin cancer. There were 677 self-reports of common cancer, excluding skin cancer. Sensitivity remained relatively unchanged (sensitivity (95% CI): 90.1% (87.6-92.3%)), but PPV increased to 88.6% (95% CI: 86.0-90.9%). In addition, rare cancers were considered without cervix cancer, as cervix cancer had a very low PPV. There were 96 self-reports of rare cancer, excluding cervix cancer. While sensitivity remained relatively unchanged, the PPV of the rare cancer group reporting increased to 54.2% (95% CI: 43.7-64.4%) when cervix cancer was excluded (Table 2).

Table 2 includes anatomical group and/or site-specific sensitivity and PPV. Site categories have been collapsed into anatomically related groups (demarcated in bold font) due to the small sample size in some categories. Sites within these groups that could be reported individually are shown under their respective anatomical group (in regular font). Male reproductive cancers had the highest sensitivity of all sites and groups reported (sensitivity (95% CI): 96.9% (92.8-99.0%)); most cancers in this category were prostate cancer, which had the highest site-specific sensitivity (sensitivity (95% CI): 96.8% (92.6-98.9%)). Breast cancer had the next highest sensitivity, at 95.6% (95% CI: 91.8-98.0%), followed by digestive/hepatic cancers (sensitivity (95% CI): 89.5% (82.3-94.4%)) and lymphatic cancers (sensitivity (95% CI): 89.3% (71.8-97.7%)). Breast cancer had the highest PPV (PPV (95% CI): 93.8% (89.5-96.6%)), followed by male reproductive cancers (PPV (95% CI): 91.1% (85.8-94.9%)), which was, again, largely due to prostate cancer (PPV (95% CI): 90.9% (85.4-94.8%)). Melanoma had a sensitivity of 79.3% (95% CI: 66.6-88.8%) and a PPV of 50.5% (95% CI: 39.9-61.2%). However, since the criteria to include specifically melanoma and exclude basal or squamous cell skin cancer changed across surveys, this may not reflect the true self-reporting accuracy of melanoma as self-reports were not specific to it. Cervix cancer had the lowest PPV at 3.6% (95% CI: 0.4-12.5%). Next to cervix cancer, ovarian cancer had the lowest PPV (PPV (95% CI): 40.0% (16.3-67.7%)), followed by CNS/eye cancers (PPV (95% CI): 44.4% (18.7-81.3%)). Rectal cancer had the lowest sensitivity (sensitivity (95% CI): 42.1% (20.3-66.5%)).

Of the 746 participants who reported a cancer and had a cancer in the ACR (true positives), 90.5% reported the correct site, 88.1% reported correct site and ± one year of diagnosis, and 68.2% reported the correct site and correct year of diagnosis (Table 3). Common cancers were reported more accurately overall, with 97.5% of 649 true positives reporting the correct site, 95.1% reporting the correct site and ± one year of diagnosis, and 73.3% reporting the correct site and year. These percentages remained relatively unchanged when skin cancer was excluded. Rare cancers were reported less accurately. Of 54 true positives, 77.8% reported the correct site, 74.1% reported the correct site and ± one year of diagnosis, and 61.1% reported the correct site and year. Removing cervix cancer had little effect on these percentages.

### 3.2. Sensitivity and PPV excluding Participants with a Diagnosis of Cancer before Baseline

After excluding participants who had a cancer diagnosis prior to enrolment, there were 789 primary cancer diagnoses in the ACR and 939 self-reports of cancer in 30,725 ATP participants. The overall sensitivity for self-report was 93.9% (95% CI: 92.0-95.5%), and the PPV was 78.9% (95% CI: 76.2-81.5%), both slightly higher than the analysis including those with cancer history (Table 2). Reporting a common cancer had a sensitivity of 91.2% (95% CI: 88.9-93.2%) and PPV of 85.9% (95% CI: 83.2-88.3%), while reporting a rare cancer had a sensitivity of 64.6% (95% CI: 53.3-74.9%) and PPV of 35.8% (95% CI: 28.1-44.1%).

Of those who correctly reported that they had cancer, 90.4% also reported the correct site, 88.0% reported the correct site and within one year of diagnosis, and 68.3% reported the correct site and year (Table 3). The site and year of diagnosis of common cancers were reported more accurately than rare cancers. Site-specific sensitivities and PPVs slightly improved or remained unchanged when excluding those with cancer before baseline, with one exception; the PPV of blood/hematopoietic cancers decreased slightly (Table 2).

### 3.3. Factors Associated with Incorrect Cancer Status or Site Reporting

Of the 30,725 ATP participants that consented to registry linkage and had no history of cancer at baseline, there were 741 true positives, 198 false positives, and 48 false negatives for reporting a cancer diagnosis. Of the 741 true positives, 71 reported an incorrect cancer site. Predictors of false positive compared to true positive reporting and incorrect site compared to correct site reporting are presented in Table 4, adjusted for predictors significant in univariate analysis (*p* < 0.2). Education, family history, and sex were not significant predictors of false positive, false negative, or incorrect site reporting in univariate analysis (*p* > 0.2). For false negative compared to true negative reporting (*N* = 789), only smoking status was significant at *p* < 0.2 in univariate analysis. Former smokers had higher odds of not reporting a diagnosed cancer (OR (95% CI): 1.92 (0.99-3.72), *p* = 0.053) compared to nonsmokers. Current smokers also had higher odds of not reporting cancer than nonsmokers (OR (95% CI): 1.32 (0.49-3.51)), but this was not statistically significant (*p* = 0.585).

Older participants had higher odds of correctly reporting cancer than younger participants, but also had higher odds of incorrectly reporting cancer site (Table 4). Participants > 70 years of age at the time of report had the highest odds of incorrectly reporting cancer site compared to those <50 years, adjusting for smoking status and rarity of cancer site (OR (95% CI): 4.19 (1.29-13.6)). Smoking status was associated with incorrectly self-reporting cancer (Table 4). Compared to nonsmokers, former smokers had higher odds of reporting a nondiagnosed cancer (OR (95% CI): 1.59 (1.10-2.29)). Current smokers had higher odds than nonsmokers of reporting a nondiagnosed cancer (OR (95% CI): 2.05 (1.29, 3.24)). Smoking was not associated with incorrect site reporting. Finally, participants with a rare cancer had much higher odds of incorrectly reporting cancer site compared to those with a common cancer, adjusting for age and smoking status (OR (95% CI): 13.7 (7.60-24.5)) (Table 4).

In participants who reported place of birth, those born outside of Canada had slightly lower odds of reporting a cancer that is not in the registry (OR (95% CI): 0.75 (0.46, 1.23)) and of not reporting a diagnosed cancer (OR (95% CI): 0.85 (0.35, 2.06)) compared to those born in Canada (Table 4). They had slightly higher odds of reporting cancer site incorrectly (OR (95% CI): 1.36 (0.70, 2.64)). However, none of the associations between the place of birth and self-report validity were statistically significant.

## 4. Discussion

This study evaluated whether self-reported cancer diagnoses were a valid outcome measure among participants of a Canadian cohort study and compared the reporting of common and rare cancers. This study contributes to the limited information on the validity of self-reported cancer diagnosis in the Canadian population. The sensitivity and PPV for reporting overall cancer status, without considering the site, were similar to reports from the US and Australia [9, 12, 14]. PPV was lower than sensitivity; self-report was more likely to lead to misclassifying someone as having cancer when they did not (false positive) than to misclassifying someone who had cancer as not having cancer (false negative). Those that correctly reported that they had cancer were also likely to report the correct cancer site; however, the year of diagnosis was reported less accurately. This was also demonstrated in a US cohort by Bergmann et al., which found that 84% of the overall true positives also reported the correct site and correct year of diagnosis within one year [12].

Common cancers were reported more accurately overall than rare cancers and, as expected, made up a majority of cancer cases in the cohort. Breast and prostate cancer, the two most common cancers in this cohort, had the highest sensitivity and PPV. These two cancers often have high self-report validity across self-report literature [9–12]. Rare cancers, however, had a lower sensitivity than common cancers and were less likely to be captured by self-report. Participants with a rare cancer were more likely to report an incorrect site than participants who had a common cancer, suggesting that rare cancer diagnoses are not well understood by participants. A logistic regression analysis supported this hypothesis; for those that correctly reported overall cancer status, the odds of reporting the site correctly were much higher among participants with a common cancer relative to those with a rare cancer. A possible explanation for this phenomenon may be that rare cancers often have less diagnostic precision [4, 5]. Ambiguous diagnostic procedures or results may be more likely to result in an incorrect or absent self-report [10, 11]. Due to the low sensitivity and low PPV of rare cancer sites reported here, it is unlikely that self-reports of rare cancer are a valid outcome measure and cancer registry linkage is necessary to capture these cases accurately. Registry linkage also provides more specific diagnosis information. Cancer research often requires narrower site categories than used in this analysis, or further information such as histology and/or cancer stage.

Cancer registry linkage not only provides a valid diagnosis but also serves as a passive follow-up to capture cases completely. There were 3,187 total cancer diagnoses that developed in our study cohort in the ACR, but only 810 occurred within active follow-up in the ATP and had the opportunity to be self-reported in a subsequent survey. Most ATP participants with cancer diagnoses did not have the opportunity to report in an ATP follow-up survey (i.e., diagnosis occurred after a participant's last survey was filled out). Rare cancers accounted for approximately 16% of the total cases that developed in the cohort, but only 10.6% of the cases are within active follow-up. Though participants who developed both common and rare cancers were lost to active follow-up, those who developed a rare cancer were more likely to be lost. One possible explanation for the differential loss to follow-up is that participants who were diagnosed with a rare cancer may have a shorter survival time than those diagnosed with a common cancer and be less likely to report. In this cohort, 40% of participants with a rare cancer have died, while 20% of participants with a common cancer have died. For those who died, the median survival time was 3.7 years and 6 years for rare and common cancers, respectively.

Finally, relying on self-reported diagnosis of cancer for inclusion criteria assumes that participants will correctly state that they have no history of cancer at study entry. If participants are not cancer-free at baseline but still included in an etiologic study, results from this study may be biased. It is unclear why some participants did not report cancer history at baseline. One reason may be that participants did not disclose their previous cancer in order to enroll in the study, as they were aware that being cancer-free at baseline was an eligibility requirement of enrolment [17]. ATP uses the cancer registry to verify cancer history after enrollment and indicates whether a participant had a prior cancer (before baseline). However, the site of cancer(s) is not disclosed and whether cancer self-report(s) in the future survey(s) are an incident or previous diagnosis is not clear. Linkage to cancer registry provides more information on these individuals and their diagnosis for researchers whose results may be impacted by cancer diagnoses before baseline.

Cancer registry linkage improved the utility of the ATP cohort by allowing for valid, detailed, and complete cancer diagnosis data. Through the partnership of ATP and the five other regional cohorts, CanPath allows for a larger sample size and further exploration into rare cancers. Although most CanPath participants have consented to linkage with cancer registry, these agreements are made within the regional cohorts [7], and administrative data cannot cross provincial boundaries without further data agreements [15]. Therefore, nationally linked data can only be obtained by applying separately for data access and registry linkage to each of the six regional cohorts. Easing access to nationally linked data would allow for better utilization of the potential CanPath has to offer in the study of rare cancers. Alternatively, allowing regional cohorts to pass along validated, and perhaps more detailed, cancer diagnosis data to the CanPath would limit the reliance on self-reported cancer diagnosis.

Using the ACR as a gold standard strengthens this analysis due to the demonstrated completeness and accuracy in reporting [18], though the possibility remains that a true cancer case was not recorded in the ACR, resulting in a false positive. There are several other limitations in this analysis. Firstly, a lack of diagnoses within active follow-up did not allow for the separate reporting of some individual sites (e.g., stomach, small intestine, liver), but general anatomical sites were still reported (e.g., digestive/hepatic). Secondly, excluding nonmelanoma skin cancer and behavior 2 cervix and prostate cancer, as per CCR reporting guidelines, likely impacted the estimated sensitivity and/or PPV of these sites. Self-reports of skin cancer were only included in the analysis if “melanoma” was specified. This likely underestimated true self-reported melanoma cases. Cervical cancer had a very low PPV; there were few (<10) ACR diagnoses of behavior 3 cervical cancer, but there were many self-reports (false positive). These self-reports were likely due to the large number of behavior 2 cervix cancer cases in this cohort (ACR, *n* = 455), many of which occurred before the baseline. Finally, the researchers should use caution in generalizing these results to the Canadian population. Participants who enroll and are followed up in this cohort are likely healthier or more health conscious than the general population [17], which could introduce a “healthy volunteer effect” [22]. However, these results are likely to be generalizable to other large observational cohorts with similar aims in health research.

## 5. Conclusions

While self-reported diagnosis is reasonably valid for some common cancer types, other cancer types, particularly rare cancers, require registry linkage to be captured completely and accurately. In order to minimize bias and loss of follow-up in the use of cohort data for rare cancer research, linkage to cancer registry is necessary. Efforts to remove barriers to cross-provincial data sharing in Canada are ongoing and are needed to allow researchers to conduct valuable research on rare cancers that these national cohorts and registries offer.

## Figures and Tables

**Table 1:** Percentage of Alberta's Tomorrow Project Health and Lifestyle Questionnaire Survey (baseline) participants that responded to each follow-up survey.

Survey	Year(s) of distribution^a^	% of baseline^b^
Health and Lifestyle Questionnaire (HLQ)	2000-2008	100
Survey 2004 (S04)	2004	30
Survey 2008 (S08)	2008	67
Updated-HLQ (UHLQ)	2009-2011	10
CORE	2011-2015	25

^a^ Years of distribution indicates the years the survey was sent out or administered; surveys could have been completed and sent in for some time after distribution. ^b^ Rounded to the nearest whole percentage. Baseline participants are those who completed the Health and Lifestyle Questionnaire (HLQ). Participants could respond to a later follow-up survey if they did not complete a previous survey (i.e., a participant could complete S08 if they did not complete S04).

**Table 2:** Sensitivity and positive predictive value (PPV) of self-reported cancer diagnoses.

Cancer type^a^	Including those with cancer diagnosis before baseline^b^			Excluding those with cancer diagnosis before baseline^b^		
	# ACR^c^	Sensitivity (95% CI)^d^	PPV (95% CI)^e^	# ACR^c^	Sensitivity (95% CI)^d^	PPV (95% CI)^e^
**Overall**	**810**	**92.1 (90.0, 93.9)**	**77.8 (75.0, 80.4)**	**789**	**93.9 (92.0, 95.5)**	**78.9 (76.2, 81.5)**

**Common**	**724**	**89.6 (87.2, 91.8)**	**84.5 (81.7, 87.0)**	**707**	**91.2 (88.9, 93.2)**	**85.9 (83.2, 88.3)**
Common (no skin)	666	90.1 (87.6, 92.3)	88.6 (86.0, 90.9)	649	91.8 (89.5, 93.8)	89.8 (87.2, 92.0)
**Rare**	**86**	**62.8 (51.7, 73.0)**	**35.8 (28.1, 44.0)**	**82**	**64.6 (53.3, 74.9)**	**35.8 (28.1, 44.1)**
Rare (no cervix)	84	61.9 (50.7, 72.3)	54.2 (43.7, 64.4)	80	63.8 (52.2, 74.2)	54.8 (44.2, 65.2)

**Oral/respiratory**	**41**	**63.4 (46.9, 77.9)**	**60.5 (44.4, 75.0)**	**36**	**69.4 (51.9, 83.7)**	**61.0 (44.5, 75.8)**
Lung and bronchus	25	76.0 (54.9, 90.6)	76.0 (54.9, 90.6)	23	82.6 (61.2, 95.0)	76.0 (54.9, 90.6)
**Digestive/hepatic**	**114**	**89.5 (82.3, 94.4)**	**85.7 (78.1, 91.5)**	**110**	**91.8 (85.0, 96.2)**	**87.1 (79.6, 92.6)**
Large intestine	68	85.3 (74.6, 92.7)	79.5 (68.4, 88.0)	65	87.7 (77.2, 94.5)	81.4 (70.3, 89.7)
Rectum	19	42.1 (20.3, 66.5)	80.0 (44.4, 97.5)	19	42.1 (20.3, 66.5)	80.0 (44.4, 97.5)
**Blood/hematopoietic**	**52**	**75.0 (61.1, 86.0)**	**86.7 (73.2, 94.9)**	**48**	**79.2 (65.0, 89.5)**	**86.4 (72.6, 94.8)**
**Skin (melanoma)**	**58**	**79.3 (66.6, 88.8)**	**50.5 (39.9, 61.2)**	**58**	**79.3 (66.6, 88.8)**	**52.9 (41.9, 63.7)**
**Breast**	**204**	**95.6 (91.8, 98.0)**	**93.8 (89.5, 96.6)**	**199**	**97.5 (94.2, 99.2)**	**94.2 (90.0, 97.0)**
**Female reproductive**	**67**	**88.1 (77.8, 94.7)**	**46.8 (37.9, 55.9)**	**66**	**89.4 (79.4, 95.6)**	**47.6 (38.5, 56.7)**
Cervix uteri	—	—	3.6 (0.4, 12.5)	—	—	3.6 (0.4, 12.5)
Uterus	49	85.7 (72.8, 94.1)	79.2 (65.9, 89.2)	48	87.5 (74.8, 95.3)	82.4 (69.1, 91.6)
Ovary	—	—	40.0 (16.3, 67.7)	—	—	40.0 (16.3, 67.7)
**Male reproductive**	**159**	**96.9 (92.8, 99.0)**	**91.1 (85.8, 94.9)**	**157**	**97.5 (93.6, 99.3)**	**93.3 (88.3, 96.6)**
Prostate gland	154	96.8 (92.6, 98.9)	90.9 (85.4, 94.8)	152	97.4 (93.4, 99.3)	93.1 (88.0, 96.5)
**Urinary**	**51**	**82.4 (69.1, 91.6)**	**87.5 (74.8, 95.3)**	**51**	**82.4 (69.1, 91.6)**	**87.5 (74.8, 95.3)**
Kidney	16	87.5 (61.7, 98.4)	73.7 (48.8, 90.9)	16	87.5 (61.7, 98.4)	73.7 (48.8, 90.9)
Urinary bladder	31	77.4 (58.9, 90.4)	85.7 (67.3, 96.0)	31	77.4 (58.9, 90.4)	85.7 (67.3, 96.0)
**CNS/eye**	**—**	**—**	**44.4 (18.7, 81.3)**	**—**	**—**	**—**
**Endocrine**	**20**	**70.0 (45.7, 88.1)**	**87.5 (61.7, 98.4)**	**20**	**70.0 (45.7, 88.1)**	**87.5 (61.7, 98.4)**
Thyroid	20	70.0 (45.7, 88.1)	87.5 (61.7, 98.4)	20	70.0 (45.7, 88.1)	87.5 (61.7, 98.4)
**Lymphatic**	**28**	**89.3 (71.8, 97.7)**	**69.4 (51.9, 83.7)**	**28**	**89.3 (71.8, 97.7)**	**69.4 (51.9, 83.7)**
**Other** ^**f**^	**—**	**—**	**—**	**—**	**—**	**—**

PPV=Positive predictive value, ACR = Alberta Cancer Registry, CI=Confidence interval, CNS=Central nervous system. ^a^Bolded groups generated by combining appropriate SEER 2018 site categories. Unbolded types are specific groups within the bolded groups above. See Table S1 for groupings. Only SEER 2018 cancer sites with >10 ACR diagnoses and/or >10 self-reported diagnoses were reported. ^b^A participant had a diagnosis before baseline if their age of first cancer diagnosis in the ACR was before their age at baseline. ^c^Number of diagnoses in the ACR. A “—“ indicates there was <10. Common and rare diagnoses add up to overall. Bolded cancer site types do not add up to overall as the “Other” and “CNS/eye” groups are not included. ^d^Sensitivities for groups with 10 or more ACR diagnoses are reported. A “—” indicates there were <10 ACR diagnoses. ^e^ PPV's for groups with 10 or more self-reported diagnoses are reported. A “—” indicates there were <10 self-reported diagnoses. ^f^Includes SEER 2018 site categories of unknown, ill-defined, bones and joints, connective and soft tissue, and retroperitoneum and peritoneum.

**Table 3:** Self-reported cancer site and year of diagnosis accuracy among ATP participants who correctly report overall, common, and rare cancer status.

	Including those with cancer diagnoses before baseline^a^				Excluding those with cancer diagnoses before baseline^a^			
	% TP's that also have correct				% TP's that also have correct			
	# TP's	Site only	Site and year ± 1	Site and year	**#** TP's	Site only	Site and year ± 1	Site and year
Common	649	97.5	95.1	73.3	645	97.5	95.0	73.5
Common (excl. skin)	600	97.8	95.5	73.7	596	97.8	95.5	73.8
Rare	54	77.8	74.1	61.1	53	77.4	73.6	60.4
Rare (excl. cervix)	52	76.9	73.1	61.5	51	76.5	72.5	60.8

Overall^b^	746	90.5	88.1	68.2	741	90.4	88.0	68.3

ATP: Alberta's Tomorrow Project; TP: true positive; excl.: excluding. ^a^ A participant had a diagnosis before baseline if their age of first cancer diagnosis in the ACR was before their age at baseline. ^b^ Overall TP does not equal common TP plus rare TP. An overall TP reported cancer and had cancer in the ACR, regardless of type. A participant with a common cancer in the ACR had to report a common cancer in order to be a common TP. Similar criterion defines a rare cancer TP.

**Table 4:** Factors associated with false positive and incorrect site self-reporting.

	Report cancer incorrectly vs. correctly (FP vs. TP)		Report site incorrectly vs. correctly (TP incorrect vs. TP correct)	
	*N* = 939		*N* = 741	
Variable	Adjusted OR (95% CI)	*p* value	Adjusted OR (95% CI)	*p* value
Age at report				
<50	1.0		1.0	
50 to <60	0.54 (0.34, 0.85)	0.008	2.52 (0.80, 7.99)	0.116
60 to <70	0.29 (0.18, 0.45)	<0.001	1.23 (0.38, 3.96)	0.732
≥70	0.35 (0.20, 0.59)	<0.001	4.19 (1.29, 13.6)	0.017
Smoking				
Never	1.0		1.0	
Former	1.59 (1.10, 2.29)	0.013	0.80 (0.43, 1.48)	0.478
Current	2.05 (1.29, 3.24)	0.002	1.48 (0.68, 3.20)	0.325
ACR diagnosis type				
Common			1.0	
Rare	N/A		13.7 (7.60, 24.5)	<0.001

FP: false positive; TP: true positive; FN: false negative; OR: odds ratio; NS: not significant; N/A: not applicable; ACR: Alberta Cancer Registry.

## Data Availability

Data can be made available upon request and in compliance with the ATP's Disclosure Policy. Information on accessing ATP's data can be found at https://myatpresearch.ca/.

## Supplementary Materials

Supplementary Materials Supplementary Table 1 includes the SEER 2018 cancer categories and corresponding ICD-O-3 topography codes used to generate the categories, the corresponding self-report accuracy type, and the combined groupings used for reporting purposes in this manuscript. Supplementary Table 2 describes the definitions used for a true positive, true negative, false positive, false negative for the overall cancer analysis, cancer site analysis, and common or rare cancer analysis.
