# Supplementary material for: The Importance of Cancer Registry Linkage for Studying Rare Cancers in Prospective Cohorts
Source: J Cancer Epidemiol. 2020 Nov 25;2020:2895276. doi: 10.1155/2020/2895276 (PMC7718062; doi:10.1155/2020/2895276)
Supplement: Supplementary Materials — Supplementary Table 1 includes the SEER 2018 cancer categories and corresponding ICD-O-3 topography codes used to generate the categories, the corresponding self-report accuracy type, and the combined groupings used for reporting purposes in this manuscript. Supplementary Table 2 describes the definitions used for a true positive, true negative, false positive, false negative for the overall cancer analysis, cancer site analysis, and common or rare cancer analysis. [file 2895276.f1.pdf]

SUPPLEMENTARY TABLES

Table S1. SEER 2018 cancer categories based on ICD-O-3 topography, corresponding self-report accuracy type, and combined groupings for reporting purposes.

ICD_0-3 (Cxxx) Topography	SEER 2018 Cancer Type(ref)	Self-report Accuracy Type	Combined Groups
C000-C006, C008-C009	Lip	Lip	Oral/ Respiratory
C019-C024, C028-C029	Tongue	Tongue	
C030-C031, C039-C041,C048-C052,C058-C062, C068-C069	Gum, Floor of Mouth, & Other Mouth	Gum, Floor of Mouth, & Other Mouth	
C079-C081,C088-C089	Salivary Gland	Salivary Gland	
C090-C091,C098-C104,C108-C109	Oropharynx	Throat	
C110-C113,C118-C119	Nasopharynx		
C129-C132,C138-C139	Hypopharynx		
C140,C142,C148	Pharynx		
C150-C155,C158-C159	Esophagus	Esophagus	Digestive/ Hepatic
C160-C166,C168-C169	Stomach	Stomach	
C170-C173,C178-C179	Small Intestine	Small Intestine	
C180, C182-C189, C199	Large Intestine	Large Intestine	
C181	Appendix	Appendix	
C209	Rectum	Rectum	
C210-C212,C218	Anal Canal & Anus	Anal Canal & Anus	
C260,C268-C269	Unspecified Digest. Organs	Unspecified Digest. Organs	
C220	Liver	Liver	
C221	Intrahepatic Bile Duct	Intrahepatic Bile Duct	
C239-C241,C248-C249	Gallbladder & Extrahepatic Bile Duct	Gallbladder & Extrahepatic Bile Duct	
C250-C254,C257-C259	Pancreas	Pancreas	
C300	Nasal Cavity (Including Nasal Cartilage)	Nasal Cavity (Including Nasal Cartilage)	Oral/ Respiratory
C301, C310-C313,C318-C319	Accessory, Sinuses, Middle & Inner Ear	Accessory, Sinuses, Middle & Inner Ear	
C320-C323,C328-C329	Larynx	Larynx	
C339	Trachea	Trachea	
C340-C343,C348-C349	Lung & Bronchus	Lung & Bronchus	
C379	Thymus	Thymus	
C380	Heart	Heart	
C381-C383, C388	Mediastinum	Mediastinum	
C384	Pleura	Pleura	
C390,C398-C399	Respiratory, NOS	Respiratory, NOS	
C400-C403,C408-C414,C418-C419	Bones & Joints	Bones & Joints	Other
C420, C421, C424	Blood, Bone Marrow & Hematopoietic System	Blood, Bone Marrow & Hematopoietic System	Blood/ Hemato-poietic
C422	Spleen	Spleen	
C423	Reticulo-Endothelial	Reticulo-Endothelial	
C440-C449	Skin-US SEER Definition	Skin-US SEER Definition	Skin
C470-C476,C478-C479,C490-C496,C498-C499	Connective & Soft Tissue	Connective & Soft Tissue	Other

C480-C482,C488	Retroperitoneum & Peritoneum	Retroperitoneum & Peritoneum	
C500-C506,C508-C509	Breast	Breast	Breast
C510-C512,C518, C529	Vagina & Labia	Vagina & Labia	Female Reproductive
C519	Vulva, NOS	Vulva, NOS	
C530-C531,C538-C539	Cervix Uteri	Cervix Uteri	
C540-C543,C548-C549	Corpus Uteri	Uterus/Endometrial	
C559	Uterus, NOS		
C569	Ovary	Ovary	
C570-C574,C577-C579	Other Female Genital	Other Female Genital	
C589	Placenta	Placenta	
C600-C602,C608-C609, C632	Penis & Scrotum	Penis & Scrotum	Male Reproductive
C619	Prostate Gland	Prostate Gland	
C620-C621,C629	Testis	Testis	
C630, C631, C637-C639	Epididymis, Spermatic Cord, Male Genital, NOS	Epididymis, Spermatic Cord, Male Genital, NOS	
C649	Kidney	Kidney	Urinary
C659, C669	Renal Pelvis, Ureter	Renal Pelvis, Ureter	
C670-C679	Urinary Bladder	Urinary Bladder	
C680-C681,C688-C689	Other Urinary Organs	Other Urinary Organs	
C690-C691, C693, C695-C698	Orbit & Lacrimal Gland (Excl. Retina, Eye, NOS)	Orbit & Lacrimal Gland (Excl. Retina, Eye, NOS)	CNS/Eye
C692	Retina	Retina	
C694	Eyeball	Eyeball	
C699	Eye, NOS	Eye, NOS	
C700-C701,C709	Meninges (Cerebral, Spinal)	Meninges (Cerebral, Spinal)	
C710-C714, C717-C719, C720-C725	Brain, Cranial Nerves, & Spinal Cord (Excl. Ventricle,	Brain, Cranial Nerves, & Spinal Cord (Excl. Ventricle,	
C715	Ventricle	Ventricle	
C716	Cerebellum	Cerebellum	
C728-C729	Nervous	Nervous	
C739	Thyroid	Thyroid	Endocrine
C740-C741,C749	Adrenal Glands	Adrenal Glands	
C750	Parathyroid	Parathyroid	
C751	Pituitary Gland	Pituitary Gland	
C753	Pineal Gland	Pineal Gland	
C754-C755,C758-C759	Other Endocrine Glands	Other Endocrine Glands	
C760-C768	Ill-Defined	Ill-Defined	Other
C770-C775,C778-C779	Lymph Nodes	Lymph Nodes	Lymphatic
C809	Unknown	Unknown	Other
--	--	Histology Only, site unclear[a]	
--	--	Site Unclear, histology not specified[a]	
--	--	Type Missing[b]	

SEER= Surveillance, Epidemiology and End Results Program, ICD-O-3=International Classification of Diseases for Oncology, Third Edition

[a] Sites generated for self-reported cancer types that did not fit into a site category, either because they specified histology only or were unclear or too broad.

[b] Site generated for those who self-reported that they were diagnosed with cancer without giving any other information.

Table S2. Definition of terms in the analyses of self-reported cancer diagnosis validity.

Term	Type of self-report analysis		
	**Overall diagnosis (any cancer)**	**Cancer diagnosis stratified by common or rare (Y= 1 common, 0 rare)[a]**	**Site-specific cancer diagnosis (X=SEER cancer category)[b]**
**TP**	Self-reported cancer in an ATP follow-up survey and had a diagnosis of cancer in the ACR	Self-reported cancer Y in an ATP follow-up survey and had a diagnosis of cancer Y in the ACR	Self-reported cancer site X in an ATP follow-up survey and had a diagnosis of cancer X in the ACR
**TN**	Did not report cancer in an ATP follow-up survey and did not have a diagnosis of cancer in the ACR	Did not report cancer Y in an ATP follow-up survey and did not have a record of cancer Y in the ACR	Did not report cancer site X in an ATP follow-up survey and did not have a record of cancer X in the ACR
**FP**	Self-reported cancer in an ATP follow-up survey, but did not have a diagnosis of cancer in the ACR	Self-reported cancer Y in an ATP follow-up survey but did not have a record of cancer Y in the ACR (e.g. Self-reported a common cancer but ACR has a rare cancer or no cancer record)	Self-reported cancer site X in an ATP follow-up survey but did not have a record of cancer X in the ACR
**FN**	Did not report a cancer diagnosis in ATP follow-up, but had a diagnosis of cancer in the ACR	Did not report cancer Y in ATP follow-up, but had a record of cancer type Y in the ACR (e.g. no self-report of cancer or a report of common cancer, but ACR has a rare cancer)	Did not report cancer site X in ATP follow-up, but had a record of cancer X in the ACR

TP=True Positive, TN=True Negative, FP=False Positive, FN=False Negative

[a] Common or rare cancer types defined as those cancer types with an incidence rate of <15/100,000 person-years, as per Walker et al. (2020)[1].

[b] SEER=Surveillance, Epidemiology, and End Results. Description of categories in Table S1.
